# Ophthalmology

**Published:** 1899-03

**Authors:** Cyril H. Walker


### OPHTHALMOLOGY.

The use of a small electro-magnet in extracting **foreign bodies** which have penetrated the eyeball has been resorted to for more than twenty years. In 1892 Haab, acting upon the principle that "He that has the strongest magnet will obtain the best results," invented a larger and immensely more powerful instrument, which he has since further improved upon. It is now some twenty-five times as powerful as the ordinary hand electro-magnet, and weighs considerably over a hundredweight. It is pivoted on an accurately constructed stand, and is most conveniently worked with the street current. Numerous cases of its success are on record. R. Barkan[1] relates four cases in which he has used it. In one case the chip of steel had entered the corneal margin and was lodged in the vitreous, but not visible. On the eighth day after the injury the patient's eye was approached to the instrument, and the foreign body gradually came forward into the lips of the wound, so that it could be easily extracted with forceps. The lens was not injured, and in fourteen days the vision was almost perfect. He is so convinced of its utility, that he asserts that no eye clinic should be without one.

In conjunction with this advance it is important to realise the progress made in the use of the **X Rays**, more especially by Mackenzie Davidson. He has devised a very simple and ingenious apparatus especially for eye work. The Crookes tube is fixed in a slide, and after the first skiagram is taken, it is moved a certain distance along the slide before the second plate is exposed. He draws special attention to the advantage of having a tube in which the rays emanate from a very small focus, and he recommends that the terminal should be of osmium. With such an apparatus two minutes is a sufficient exposure for each plate, and it should always be possible to estimate the exact position of any piece of metal not smaller than a pin's head.

The cost of eye injuries has not been sufficiently recognised. The nation, to say nothing of clubs and benefit societies, is put to great unnecessary expense owing to the indifference, we might almost say the aversion, with which workmen regard **protective glasses.** In Cologne the "Quarry-Union" has taken the matter up. Dr. Hillemanns[2] finds there are defects in all the protective spectacles in use, whether of glass, quartz, mica, or gauze. They are either uncomfortable or, owing to obstructing vision, reduce a man's wage-earning power when doing piecework. He recommends goggles, consisting of a short truncated cone of open-meshed galvanized-iron gauze, the base of which fits the face accurately, whilst the summit is closed in by a disc or lens of glass or, better, rock crystal. Since the introduction of these protectors at the railway works at Cologne, five years ago, no eyes have been lost, and the men wear them willingly. Hillemanns urges the profession to direct the attention of the laity to the advantages of proper protectors.

There is not much new to record in the technique of **cataract extraction**; in fact, some of the later departures are really reversions to methods abandoned thirty or forty years ago.

[1] *Arch. Ophth.*, 1898, xxvii. 37. [2] *Ibid.*, 527.

This is mainly due to the introduction of cocaine and antiseptics rendering methods safe and practicable which were formerly often attended with failure. Prof. C. Schweigger[1] advocates extraction of the lens by means of a downward corneal flap—practically the old flap operation of one hundred years ago. This method possesses hardly any advantage, except that the corneal wound is more easily inspected after the operation. But this is rarely necessary after all. Various surgeons have attempted to suture the corneal wound ; but this procedure does not seem to ensure any better results. The most that can be said of it is that it is not so risky as would at first sight appear probable. Secondary operations (needling opaque lens capsule) are not to be lightly undertaken. Schweigger found that in no less than 15 per cent. of his cases, this operation was followed by disagreeable and tedious irritation, lasting weeks or months. This occurred even in cases where there was no traction on the ciliary body during the needling. For the last two years he has used a modification of de Wecker's forceps-scissors, and clipped through the membrane in every case of capsular cataract, and has met with good results.

Tho etiology of **pterygium** is still a matter of dispute. Fuchs regards it as intimately connected with pinguecual; others regard them as two entirely distinct conditions. Henry Lopez,[2] of Havana, has had unusual opportunities for studying the two diseases. He estimates that more than half the male population of Cuba suffer from pterygium—we presume he refers only to male adults, because in the next paragraph he says it is very rarely found before the twentieth year. He is convinced that pterygium and pinguecula are but different stages of the same disease. According to him pinguecula grows up to the corneal margin and forms a sharply defined raised lump ; foreign bodies lodge in the angle between this lump and the cornea, destroy the corneal epithelium, and render the cornea a prey to the advancing growth. He does not look upon pterygium as being particularly obstinate or necessitating the complicated operations some have devised. Avulsion of the "head" from the cornea, excision of the whole growth, and suture of the gap in the conjunctiva are effective in almost every case.

In a very suggestive paper on "**Ophthalmoscopic evidence of general arterial disease,**"[3] Marcus Gunn draws special attention to the "influence of the arterial pressure on the venous blood-stream where the artery and vein cross one another." The appearances that he alludes to are easy to see, now that attention has been drawn to the subject. At first the vein is bent, and consequently loses its central "light-streak" for a short distance on either side of the artery. Later, the venous current

1 *Arch. Ophth.*, 1898, xxvii. 255.

2 *Ibid.*, 279.

3 *Tr. Ophth. Soc. U. Kingdom*, 1898, xviii. 356.

is markedly impeded and the peripheral portion of the vein is abnormally engorged. His explanation is, that the coats of the artery have undergone a hyaline-fibroid change, the vessel is unduly rigid and tightly distended. He adds: "The result of the increased difficulty in arterial circulation will be to diminish the rapidity of the blood-stream in the capillaries and veins. As a result, there will be a tendency to the escape of *liquor sanguinis* into the surrounding tissues, and this tendency will be much increased where the venous circulation is mechanically impeded, as by an overlying artery." Thus a vicious circle is established, and we get hemorrhages and white patches in the retina. "In the most advanced cases the lines of the folds which radiate from the fovea centralis, due to the œdema, are sometimes eventually marked out by the deposit of white spots of degenerated effusion, so that we get the ophthalmoscopic appearances diagnostic of so-called albuminuric or renal retinitis." Old age does not produce these conditions, and, associated with local disease at any rate, the same appearances may be present in patients of eighteen or twenty.

It would seem that it is not improbable that the formation of this vicious circle may be a factor in some cases of so-called **retinitis proliferans,** but various diseases have been described under this term, and a very different sequence of events is probable in the series of cases mentioned by J. E. Weeks.[1] In twenty-one out of twenty-two cases that he examined after death the retinitis proliferans was due to a fibrinous exudation or hemorrhage into the vitreous, upon which vascular connective tissue creeps up from the retina, thus forming an ophthalmoscopic picture resembling the class of retinitis proliferans first alluded to.

In the *Transactions of the Ophthalmological Society* for last year there is a very elaborate report on the question of **excision of the eyeball** and allied operations, from which I extract the following. Removal of a portion of the eye (abscission) is as old as Celsus, but the first recorded case of "extirpation" was performed in 1583 by means of a spoon with cutting edges, and until 1840 a somewhat similar instrument was invariably used. In 1850 the late Augustin Prichard[2] was the first to extirpate an injured blind eye, and he then advocated excision of the exciting eye in all cases of sympathetic ophthalmitis. Evisceration, as practised by von Graefe in 1863 in cases of suppurative panophthalmitis, has not been shown to have any advantages over excision. The relative frequency of sympathetic inflammation, after the introduction of a globe of silver or glass into the emptied sclerotic (Mules's operation) is infinitely greater than after simple excision. It should only be resorted to in such cases as painful blind glaucomatous eyes, or within fourteen days of an injury in a young subject. The only

[1] *Tr. Am. Ophth. Soc.*, 1897, viii. 158. [2] *Prov. M. & S J.*, 1851, 66.

important objection to the introduction of a similar globe into Tenon's capsule is that it comes out in about a quarter of the cases, and always necessitates for the patient a longer duration in hospital—roughly, twelve days, instead of three days as in excision. Optico-ciliary neurectomy, excision of a portion of the optic and ciliary nerves through an incision over the internal rectus, is only admissible in such cases as painful blind glaucomatous eyes. It has never been known to be followed by sympathetic inflammation except in eyes which had previously been injured in a way likely to cause sympathetic ophthalmitis. It obviates the necessity of an artificial eye; but, unfortunately, the pain for which the operation was undertaken sometimes returns, and in several instances ulceration of the cornea has followed.

For **Ophthalmia neonatorum** Prof. Credé, in 1882, advocated as a routine treatment the instillation of a few drops of a 2 per cent. solution of nitrate of silver into the eyes as soon as the child's head was born. Lucien Howe[1] summarises the results of this treatment. The combined experience of a number of obstetricians shows that in a series of more than 40,000 cases ophthalmia neonatorum was fifteen times more prevalent where no treatment was used than when the Credé method was adopted. Solutions of corrosive sublimate, especially the stronger solutions, gave slightly better results; but they cause such excessive inflammation in infants, that the advantage is on the whole in favour of nitrate of silver. He thinks that the Credé method should be made compulsory in all public institutions. Inquiries at 110 of the largest German clinics resulted in the discovery of only four cases in which the method was supposed to have done harm, and in some of these it was doubtful if the nitrate of silver was to blame. Protargol, a protein compound containing 8.3 per cent. of silver, is recommended by Adolf Alt[2] as a substitute for nitrate of silver. It is readily soluble in hot or cold water, and not precipitated by albumin or chloride of sodium. It consequently penetrates much more readily into all the crevices of the conjunctival sac than silver nitrate solution, and its application is much less painful. He uses a 1 per cent. solution, but others have found a 5 per cent. solution more effectual. Largin is another somewhat similar compound which has recently been introduced. Mr. H. G. Parker, the house-surgeon at the Bristol Eye Hospital has obtained good results with a 5 per cent. solution, and judging by the results of a few cases its use can be recommended in all cases of conjunctivitis attended with purulent discharge.

CYRIL H. WALKER.

[1] *Tr. Am. Ophth. Soc.*, 1897, viii. 52; *Am. J. Ophth.*, 1898, xv. 80.
[2] *Am. J. Ophth.*, 1898, xv. 22.

